# Lipoma with osteocartilaginous metaplasia in infrapatellar fat pad: A case report and review of literature

**DOI:** 10.1097/MD.0000000000031303

**Published:** 2022-10-21

**Authors:** Segi Kim, Cheungsoo Ha, Ah-Young Kwon, Wonchul Choi

**Affiliations:** a Department of Orthopaedic Surgery, CHA Bundang Medical Center, CHA University School of Medicine, Gyeonggi-do, Republic of Korea; b Department of Pathology, CHA Bundang Medical Center, CHA University School of Medicine, Gyeonggi-do, Republic of Korea.

**Keywords:** chondrolipoma, infrapatellar fat pad, lipoma, osteolipoma

## Abstract

Lipomas are tumors composed of mature adipocytes, originating from the mesoderm, and are the most common soft tissue tumor. According to the World Health Organization classification of human soft tissue and bone tumors, there are 14 types of benign tumors, including mature adipose tissue. Osteolipoma is known as the rarest subtype of lipoma.

**Patient concerns:**

A 63-year-old female presented to our hospital for the evaluation and treatment of a palpable mass with pain in the right knee.

**Diagnosis:**

The diagnosis was confirmed as lipoma with osteocartilaginous metaplasia.

**Interventions:**

Surgical removal of the tumor was performed.

**Outcomes:**

The main symptoms improved immediately after the surgery and recovered without any complications or recurrence until 2 years after surgery.

**Lessons:**

Lipoma with osteochondral degeneration is a rare variant of lipoma and it is important to differentiate it from other malignant tumors. Pathological confirmation should be performed after marginal resection of the mass.

## 1. Introduction

A lipoma is a tumor composed of mature adipocytes, originating from the mesoderm, and is the most common soft tissue tumor.^[1]^ Histologically, a membrane made of fibrous tissue separates the periphery and the border of lipoma, and it is filled with mature adipocytes and cells of mesoderm origin.^[1]^ Lipomas can occur in any part of the body, mainly in the subcutaneous tissue of the neck, back, and extremities.^[1,2]^ Normal lipoma consists only of mature adipocytes, but there are several variants depending on the other components of it.^[1,2]^ The World Health Organization classification of human soft tissue and bone tumors describes 14 types of benign lipomas containing mature adipose tissue.^[3]^ Among them, osteolipma is known as the rarest subtype of lipoma, and the first case was reported in 1959.^[3]^ Lipomas containing both bone and cartilage degeneration are even rarer.^[3]^ Lipomas formed around the knee joint are mostly observed through follow-up, but if they cause tenderness or mechanical symptoms such as joint movement disorders, they may be surgically removed and also removed for cosmetic purposes.^[1,2]^ In addition, it is important to differentiate these lipomas from other malignant or benign soft tissue tumors containing bone tissue components.^[2]^ The authors would like to report a case of lipoma with osteochondral degeneration that occurred in the infrapatellar fat pad of the knee, along with a literature review.

## 2. Method

### 2.1. Ethical approval

This study was approved by the Institutional Review Board of CHA Bundang Medical Center (CHAMC 2022-08-028) at the CHA University School of Medicine (Korea). This study was conducted in accordance with the principles of the 1975 Declaration of Helsinki (revised in 2008).

We obtained patients’ clinical and radiological data. Informed written consent was obtained from the patient for publication of this case report and accompanying images.

### 2.2. Data access

A systematic literature review was conducted using 3 different databases (PubMed, Scopus, and Web of Science) using the keywords and search terms “Osteolipoma” OR “Chondrolipoma.”

## 3. Case report

A 63-year-old female patient visited our outpatient clinic with pain in the right knee joint that occurred approximately 1 year ago without any particular trauma. Conservative treatment, including drug treatment, was administered at another hospital. However, symptoms did not improve, and computed tomography performed at another hospital revealed a mass within the infrapatellar fat pad.

**Figure 1.**
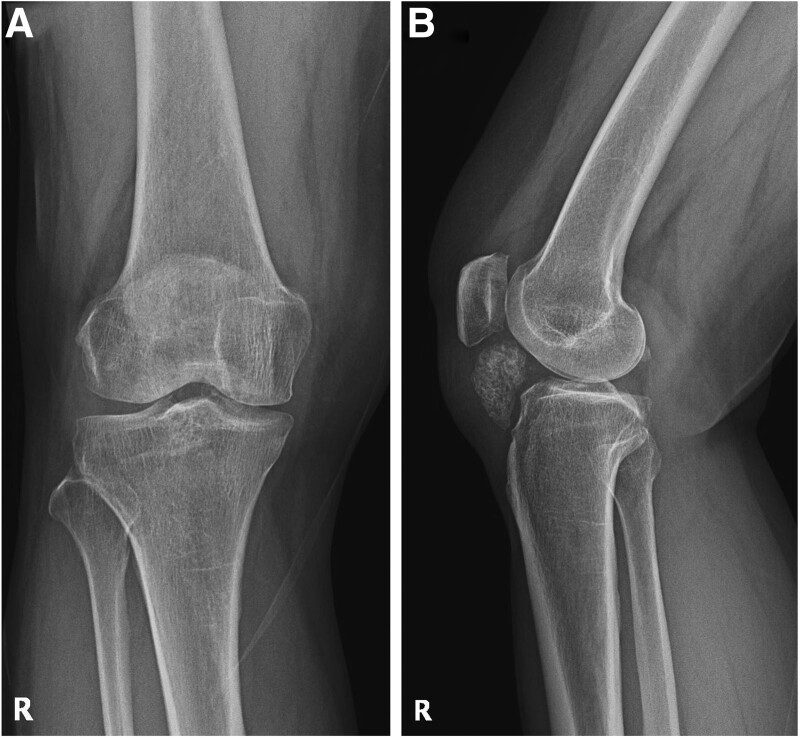
The simple radiography images show a mass with calcification in the infrapatellar fat pad of the right knee. AP view (A), Lateral view (B).

**Figure 2.**
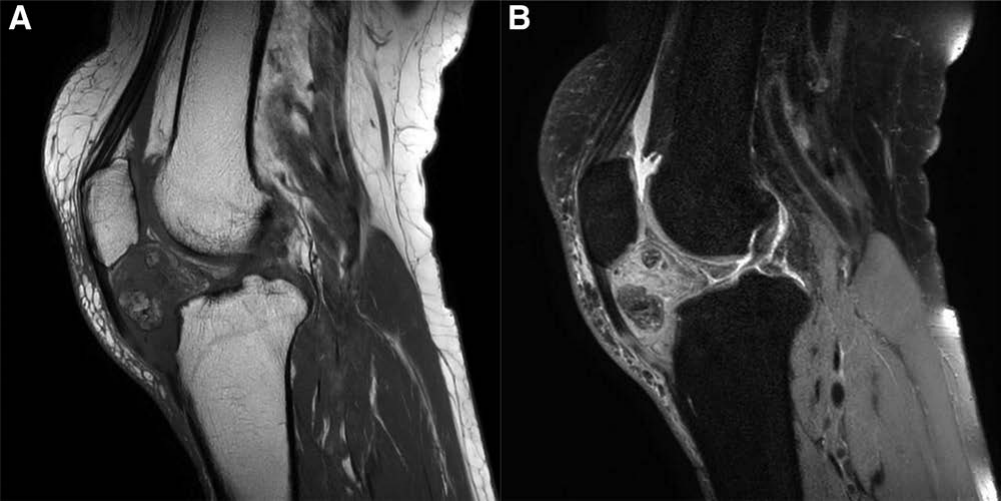
Magnetic resonance imaging (MRI) shows clearly the mass in the infrapatellar fat pad. It shows a lobulated mass. T1 image (A), T2 image (B).

On physical examination performed at our hospital, a palpable mass accompanied by tenderness was observed on the anterior part of the right patellar tendon. The range of motion was limited to 30° of flexion and 130° of further flexion. The blood test was normal, and a simple radiographic examination revealed a lesion suspected of being a 3.4 × 2.1 cm ossified mass inside the infrapatellar fat pad (Fig. 1). Magnetic resonance imaging examination was performed, and a 5.7 × 2.5 × 3.8 cm sized, irregular shading-enhancing mass was observed in the anterior region of the inner infrapatellar fat pad in the space between the tibial plateau and the patellar tendon. The interior of the mass was irregular, and multiple osteochondral bodies of various sizes were present (Fig. 2). Osteolipoma, chondrolipoma, or degenerated lipoma should be differentiated through imaging, and surgery was planned for mass removal and biopsy.

**Figure 3.**
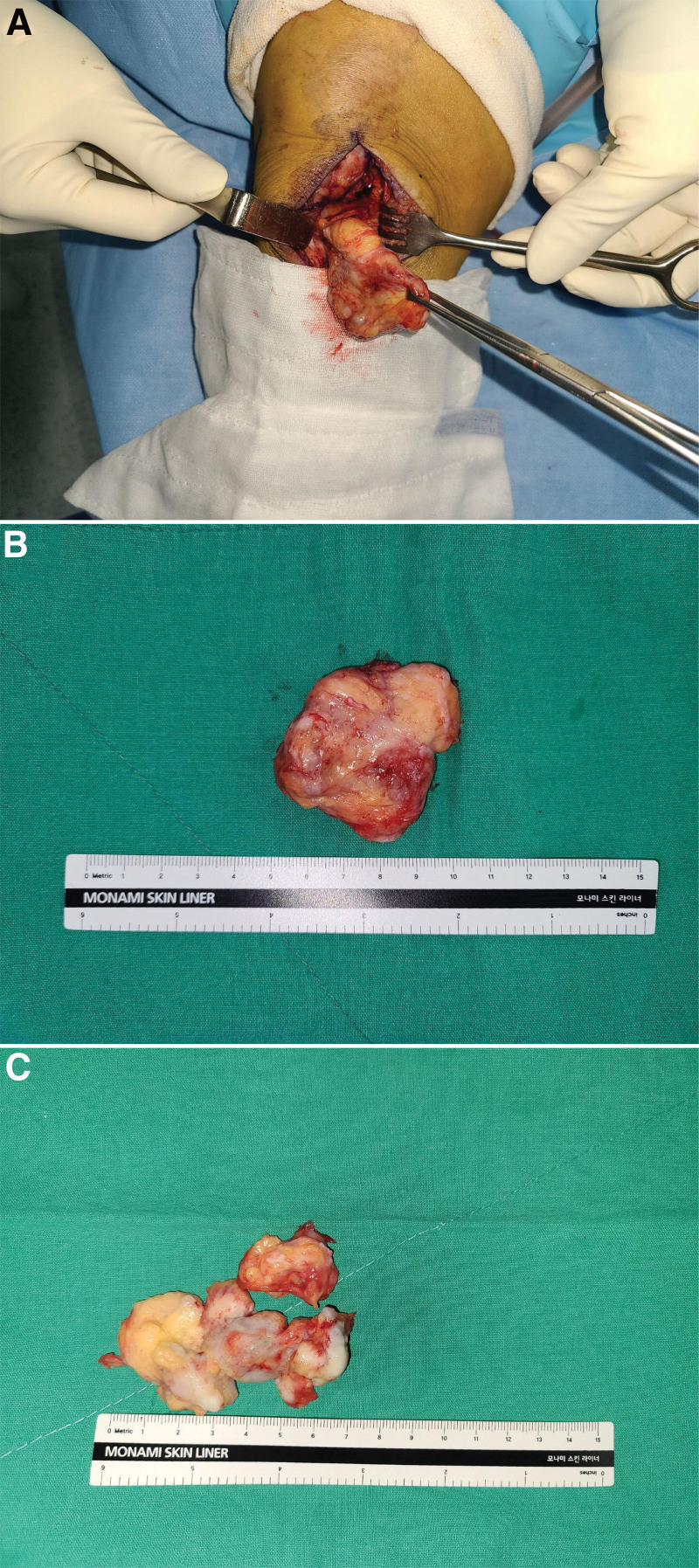
The tumor was excised (A), A pathologic specimen of lipoma with osteocartilaginous metaplasia (B and C).

**Figure 4.**
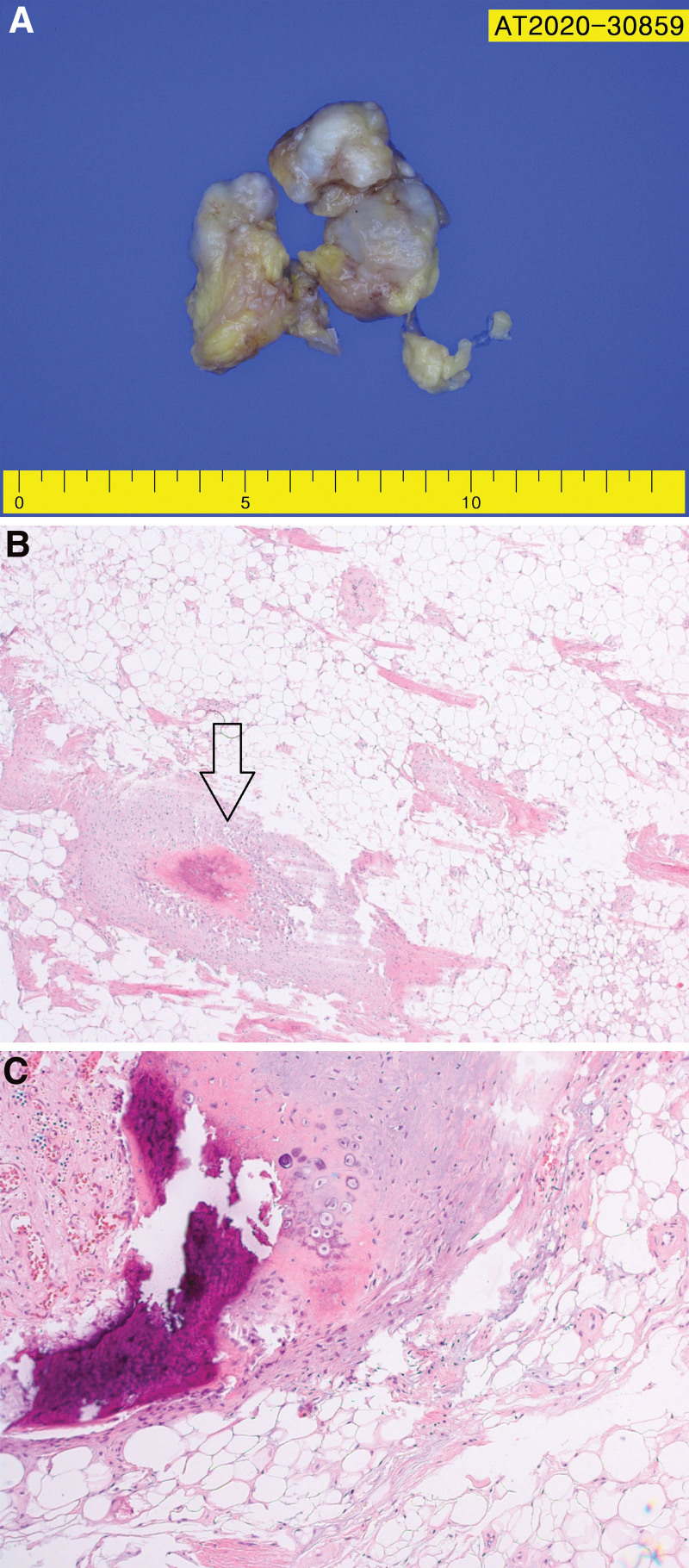
Gross and microscopic features of tumor. The tumor is an encapsulated lobulating mass (A), The tumor consists of mature adipocytes with focal osteoid and chondroid metaplasia (arrow) (H-E stain, ×40) (B), Hyaline cartilage with ossification and micro-calcification is shown within mature adipocytes. Cytologic atypia is not shown (H-E stain, ×100) (C).

Under general anesthesia, a capsule-enclosed mass in the infrapatellar fat pad was identified through the medial approach of the patella to the joint cavity. The mass was completely separated from the surrounding tissues, and marginal excision was performed. The extracted mass was approximately 5.1 × 2.2 × 4.0 cm in size. The interior of the mass was composed of bone and cartilage tissues of various sizes and irregular shapes (Fig. 3). Macroscopically, the mass was in the form of lobules, well-demarcated and encapsulated. The excised surface contained small, white, ossified tissues with a uniform yellow cross-section. The mass was histologically composed of benign adipocyte tissue, and bone and cartilage tissues of various sizes and irregular shapes were observed between the adipocytes. The cells constituting these bone and cartilage tissues also showed benign findings without cytological atypia (Fig. 4). Simple radiographic examination performed immediately after surgery confirmed that the ossified lesion was completely removed. The final pathological result confirmed a lipoma with osteocartilaginous metaplasia.

Joint movement and weight-bearing were allowed immediately after the operation. Anterior knee joint tenderness and flexion restriction improved immediately after the operation and recovered without any complications related to surgery. It was confirmed that daily life was possible without recurrence until 2 years after surgery.

## 4. Discussion

Although benign lipomatous lesions are common, it is difficult to determine the exact frequency of incidence because they are difficult to recognize clinically or are observed even though they are recognized.^[2]^ The cause of lipoma degeneration is not yet clear, but there are claims that it is caused by chronic stimulation or trauma.^[4]^ In addition, 2 representative hypotheses exist regarding the origin of the osteochondral degeneration of lipoma. First oncogenic mutations occur in various mesenchymal cells that directly differentiate into adipocytes, osteoblasts, and chondroblasts simultaneously. The second hypothesis is that secondary neoplastic mutations gradually occur within the lipoma, and undifferentiated mesenchymal cells in the stroma differentiate into chondroblasts and osteoblasts.^[4]^ In recent years, chromosomal rearrangement abnormalities have been observed in lipoma degeneration, and further research on chromosomal abnormalities as the cause of the occurrence is needed. In addition, it is argued that there is also the influence of growth factors such as transforming growth factor-β, latent transforming growth factor-β binding protein-1, and bone morphogenesis protein.^[5]^

The infrapatellar fat pad is an intra-capsular and extra-synovial structure. Common neoplastic lesions occurring in the infrapatellar fat pad include peri-articular chondroma or osteochondroma, focal pigmented villonodular synovitis, synovial lipoma, synovial chondromatosis, and synovial hemangioma.^[6]^ This case is a lipoma with osteochondral degeneration, which is a variant of a lipoma that occur in the infrapatellar fat pad. Lipoma with osteochondral degeneration is a rare variant, most of which has been reported in the head and neck. One case has been reported in the right hip in Korea.^[7]^ Two cases of infrapatellar fat pad have been reported worldwide,^[8,9]^ but no such case has been reported in Asia.

Lipoma accompanied by osteochondral degeneration can be confirmed as a calcified mass on simple radiographic examination, but these findings are also observed in chondroma, enchondroma, chondrosarcoma, and liposarcoma.^[10]^ It is difficult to confirm the diagnosis with a simple radiographic examination alone.^[10]^ In particular, it is important to differentiate lipoma from soft tissue malignancies such as liposarcoma and osteosarcoma.^[10]^ In the study by Gaskin and Helms, among the tumors suspected of liposarcoma on magnetic resonance imaging, it was reported that 64% of them were benign lipoma variants, and 18% of them were osseous or cartilaginous lipomas.^[10]^ Therefore, the diagnosis of lipoma accompanied by osteochondral degeneration should be made through pathological examination to differentiate it from malignant lesions. The absence of cytological atypia is a distinguishing point from malignant tumors.^[4]^ In the pathological findings of this patient, bone and cartilage tissue without cytological atypia was observed in the tumor composed of benign adipocytes.

## 5. Conclusion

Lipoma with osteochondral degeneration is a rare variant of lipoma that rarely occurs in the lower extremities. We encountered a case of lipoma accompanied by osteochondral degeneration that occurred in the infrapatellar fat pad, and the final diagnosis was made by pathologic examination after marginal resection. The patient had no recurrence until 2 years after the final diagnosis.

## Author contributions

Conceptualization: Wonchul Choi.

Data curation: Segi Kim, Cheungsoo Ha.

Formal analysis: Segi Kim, Cheungsoo Ha.

Investigation: Segi Kim, Cheungsoo Ha.

Methodology: Segi Kim, Cheungsoo Ha.

Project administration: Wonchul Choi.

Writing – original draft: Segi Kim, Cheungsoo Ha, Ah-Young Kwon.

Writing – review & editing: Wonchul Choi.
